# Uncorrelated bilateral cortical input becomes timed across hippocampal subfields for long waves whereas gamma waves are largely ipsilateral

**DOI:** 10.3389/fncel.2023.1217081

**Published:** 2023-07-27

**Authors:** Sara Hernández-Recio, Ricardo Muñoz-Arnaiz, Víctor López-Madrona, Julia Makarova, Oscar Herreras

**Affiliations:** ^1^Laboratory of Experimental and Computational Neurophysiology, Department of Translational Neuroscience, Cajal Institute, CSIC, Madrid, Spain; ^2^Program in Neuroscience, Autónoma de Madrid University-Cajal Institute, Madrid, Spain; ^3^INSERM, INS, Inst Neurosci Syst, Aix Marseille Univ, Marseille, France

**Keywords:** bilateral circuits, cortico-hippocampal circuits, interhemispheric correlation, field potential generators, network oscillations

## Abstract

The role of interhemispheric connections along successive segments of cortico-hippocampal circuits is poorly understood. We aimed to obtain a global picture of spontaneous transfer of activity during non-theta states across several nodes of the bilateral circuit in anesthetized rats. Spatial discrimination techniques applied to bilateral laminar field potentials (FP) across the CA1/Dentate Gyrus provided simultaneous left and right readouts in five FP generators that reflect activity in specific hippocampal afferents and associative pathways. We used a battery of correlation and coherence analyses to extract complementary aspects at different time scales and frequency bands. FP generators exhibited varying bilateral correlation that was high in CA1 and low in the Dentate Gyrus. The submillisecond delays indicate coordination but not support for synaptic dependence of one side on another. The time and frequency characteristics of bilateral coupling were specific to each generator. The Schaffer generator was strongly bilaterally coherent for both sharp waves and gamma waves, although the latter maintained poor amplitude co-variation. The lacunosum-moleculare generator was composed of up to three spatially overlapping activities, and globally maintained high bilateral coherence for long but not short (gamma) waves. These two CA1 generators showed no ipsilateral relationship in any frequency band. In the Dentate Gyrus, strong bilateral coherence was observed only for input from the medial entorhinal areas, while those from the lateral entorhinal areas were largely asymmetric, for both alpha and gamma waves. Granger causality testing showed strong bidirectional relationships between all homonymous bilateral generators except the lateral entorhinal input and a local generator in the Dentate Gyrus. It also revealed few significant relationships between ipsilateral generators, most notably the anticipation of lateral entorhinal cortex toward all others. Thus, with the notable exception of the lateral entorhinal areas, there is a marked interhemispheric coherence primarily for slow envelopes of activity, but not for pulse-like gamma waves, except in the Schafer segment. The results are consistent with essentially different streams of activity entering from and returning to the cortex on each side, with slow waves reflecting times of increased activity exchange between hemispheres and fast waves generally reflecting ipsilateral processing.

## Introduction

The cortico-hippocampal circuits are critically involved in the processing of cognitive and behavioral tasks (Jurado-Parras et al., 2013; Schlesiger et al., 2015; Sugar and Moser, 2019; Alexander et al., 2020; Chao et al., 2020; Hainmueller and Bartos, 2020). Information transfer is bidirectional, with the main input coming from two closely related cortical areas, the medial and lateral entorhinal cortices (MEC, LEC), and its return to the latter from the CA1 after being processed in the hippocampal subfields and retrohippocampal structures (Steward and Scoville, 1976; Krettek and Price, 1977; Köhler et al., 1978). This canonical circuit globally marks the hippocampus as a station where sensory information is contextualized by global state variables and those affecting navigation, both motor and spatial (Vinogradova, 2001; Zeidman and Maguire, 2016; Eichenbaum, 2017; Ekstrom and Ranganath, 2018). This overall view has mainly derived from studies carried out in a single hemisphere and exploring activity in individual hippocampal subfields in relation to specific tasks or through correlational studies between two nuclei or areas. However, in addition to the classic lateralization affecting sensory-motor and language areas (Geschwind, 1979; Thomas et al., 1997; Ha et al., 2012), numerous fMRI studies have shown highly distributed and lateralized representation in the cortex for a wide variety of tasks, mainly in humans but also in rodents (Henkin and Levy, 2001; Vigneau et al., 2006, 2011; Klur et al., 2009; Iglói et al., 2010; de Celis Alonso et al., 2012; Hertrich et al., 2020; Jordan, 2020). Both the cortex and the hippocampus have interhemispheric connections whose function is assumed but comparatively poorly studied. This is an important deficiency because, from a connective point of view, a connection between ipsilateral nodes has the same value as an interhemispheric one.

In the rodent hippocampus, symmetric activities in some field potentials (FPs), such as theta rhythm and sharp waves (Sabolek et al., 2009; Valeeva et al., 2019), have been described, but these are just a minimal sample of the many and varied dynamics that can be found. Moreover, bilateral temporal coherence of these FP activities does not imply redundancy of information, as the FP is a synaptic envelope whose qualitative content cannot be assumed without specific verification in each case (Herreras, 2016). For example, the input from CA3 to CA1 manifests as chains of strongly paired gamma waves between hemispheres, with a right-side anticipatory bias that has been interpreted as the result of a coupling mechanism between autonomous natural oscillators on each side, whereas marked waveform differences indicated lateralized processing (Benito et al., 2016). In previous segments of the trisynaptic circuit, other studies have shown lateralization of granule cell (GC) firing during processing of contextual information in virtual spatial navigation (Cholvin and Bartos, 2022).

In this study, we aim to obtain a global picture of spontaneous bilateral activity flow in the different subfields of the cortico-hippocampal loop using multisite linear FPs that can be decomposed into pathway-specific synaptic activities through spatial discrimination techniques (Bell and Sejnowski, 1995; Makarov et al., 2010; Herreras et al., 2015; Whitmore and Lin, 2016). The identity of the pathways has been previously determined through a battery of tests, including unit recording, neurotransmitter blockade, directed lesions, and evoked potentials (Korovaichuk et al., 2010; Makarova et al., 2011; Fernández-Ruiz et al., 2012; Martín-Vázquez et al., 2013; Benito et al., 2014, 2016). This allows simultaneous access to fluctuations in activity in various intra-hippocampal connections, local circuits, and extrinsic afferents.

To avoid the uncertainties arising from the coexistence of multiple and strongly rhythmic generators that dominate some electrographic states (López-Madrona et al., 2020; Herreras et al., 2022), we have focused on irregular (non-theta) activity characteristic of anesthetized animals. This pattern still contains numerous rhythmic oscillations of lower frequency (Buzsáki et al., 1983; Mysin and Shubina, 2023) as well as stereotyped events of extremely variable morphology and duration (Jarosiewicz and Skaggs, 2004; Hulse et al., 2017). For this reason, a battery of tests has been used to explore different temporal and frequency aspects of the coupling between homonymous bilateral generators. An analysis of the relationships within each hemisphere has also been performed.

The most notable finding is that one of the main inputs to the hippocampus originating from the LEC is strongly lateralized during global irregular electrographic states. However, across the hippocampal subfields, the activity adopts similar dynamics on both sides for long waves in the delta-theta band, whereas the shorter (gamma-like) waves are predominantly unilateral. Detailed analysis reveals distinct dynamics in each of the FP generators in all subfields that reflect specific integration into particular populations. We interpret data in terms of the different functions and coordination needs of the bilateral and ipsilateral circuits.

## Materials and methods

The experiments were performed in accordance with EU (2010/63/UE), Spanish (RD 53/2013) and local (Autonomous Community of Madrid, Order 4/8/1988) regulations regarding the use of laboratory animals, and the experimental protocols were approved by the Research Committee of the Cajal Institute. Individuals were female Wistar rats (250–300 g) of between 3 and 4 months. For this study we selected six out of a cohort of 16 experiments that fulfilled strict functional criteria regarding bilateral recordings (see below). Animals were inbred at the local animal facilities in a 12-hour light/dark cycle, stable temperature (20–22°C), and food and water were given *ad libitum*.

### Experimental procedures and design

**FIGURE 1:**
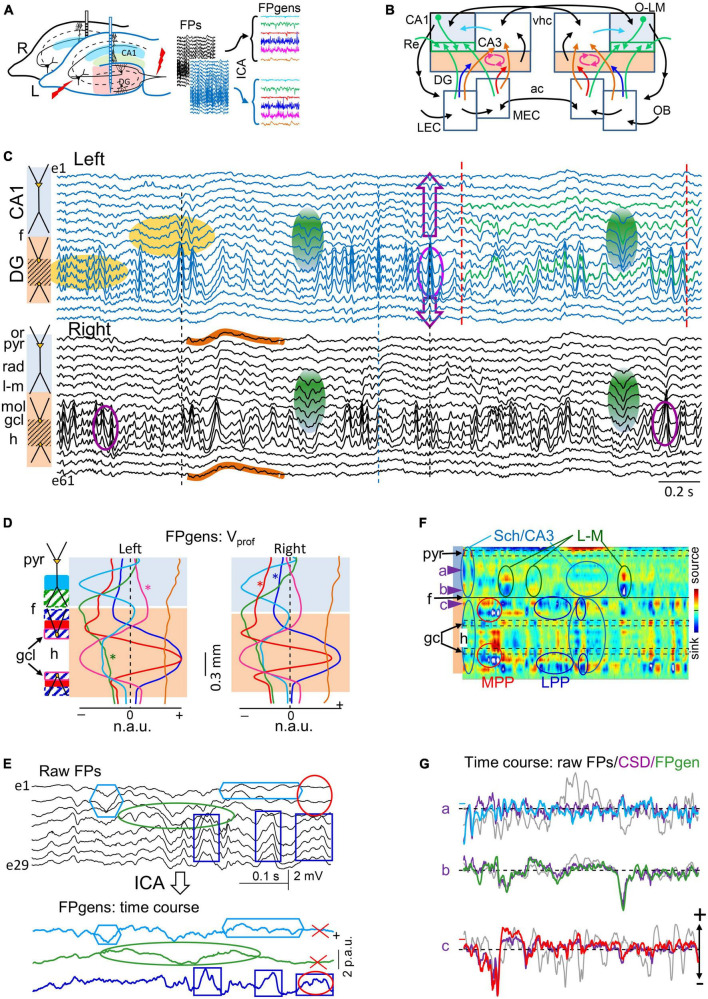
Experimental paradigm and retrieval of clean dynamics in bilateral cortico-hippocampal field potential (FP) generators. **(A)** Experimental setup and data treatment. Linear arrays were located at homotopic sites of the dorsal left and right hippocampi. Recordings were acquired simultaneously and each group was analyzed separately by an independent component analysis (ICA). Stimulating electrodes (in red) were placed at different sites to identify some FP generators through evoked potentials. Shaded areas mark the sites of maximal amplitude of the FP generators. **(B)** Scheme of some cortico-hippocampal connections relevant for the present study. Colored regions (CA1 and DG) correspond to areas recorded with linear arrays. The compounded synaptic currents of principal cells raise the FPs in these regions, which act as a screen revealing the dynamics of incoming pathways. The green boxes in CA1 highlight the st. lacunosum-moleculare (L-M), which distal position makes it prone to raise large FPs. Colored arrows mark the pathways contributing to FPs in the hippocampus and are retrievable through the ICA (color code is maintained in all figures): Sch (cyan, CA3→CA1), L-M (green, inputs to the CA1 st. L-M), LPP and MPP (blue and red, DG inputs from the lateral and medial entorhinal cortices, LEC, MEC), and GCsom (pink, input to granule cell layer (gcl). Ac, Anterior commissure; OB, olfactory bulb; O-L, or-lac; Re, nucleus reuniens; vhc, ventral hippocampal commissure. **(C)** Sample epoch of raw FPs across the CA1 and DG layers (every other electrode is plotted). Several bands of coherent voltage fluctuations are observed that indicate multiple activation in different synaptic territories (e.g., purple ovals mark alpha waves in the DG hilus, and green ovals mark large waves in the CA1 st. L-M). Spatial domains containing identifiable FP motifs may overlap partially or completely across strata, distorting each other. Orange ovals mark zones of heavy mixing in the DG hilus, and the CA1/DG border (hippocampal fissure: f). FP waves are observed with decreasing amplitude beyond their core site, and leave residues that may be confounded with local waves (DG alpha waves spread past the CA1 and into thalamus: purple arrows). Some waves have stable waveform throughout all recordings, denoting a remote (volume-conducted) origin (outlines in brown). Note that some waves/motifs are overtly bilateral (green ovals), and others are highly asymmetrical (DG alpha waves). Strata: or, oriens; pyr, pyramidale; rad, radiatum; l-m, lacunosum-moleculare; f, hippocampal fissure; mol, moleculare; h, hilus. **(D,E)** The ICA returns the spatially-coherent components, and provides each one’s spatial profile and the temporal dynamics free of a contribution by others. **(D)** A set of FP components is obtained per recording shank whose characteristic spatial distribution (V_prof_) enabled matching left and right FP generators for subsequent comparative study of time dynamics. Each displays a maximum at the respective synaptic zone in the CA1 (Sch and L-M), whereas layer folding in the DG transfers the maximum to the hilus (LPP, MPP and GCsom). The linear “tails” (asterisks) left by some generators in neighbor structures denote and quantify the spread of potentials away from their site of origin through volume conduction. Non-zero flat profiles (brown) correspond to volume-conducted contributions from sources outside the recorded area (e.g., brown curves in panel **C**). **(E)** Example of wave segregation into specific FP generators (colored boxes). Volume conducted residues (e.g., gamma waves in the red oval) are assigned to one FP generator despite being present all throughout. **(F)** Current-source density (CSD) analysis finds sinks (blue) and sources (yellow-red) of current and removes volume-conducted contributions. The contour plot corresponds to the selected epoch between dashed red lines in panel **(C)**. Numerous spatiotemporal clusters span domains where inward and outward currents can be matched to waves in known pathways (ovals), but many other surges of current are poorly mated to FP waves due to overlap of currents from different pathways in the same strata, distorting each other’s time course (magnitude and polarity). Arrowheads *a-c* point to strata where FP generators have the synaptic territory and the time course is plotted in panel **(E)**. **(G)** Superposition of time courses for a selected epoch in three sites corresponding to the strata where FP generators have a maximum. Each group contains the FP (in black), the time course of the FP generator (color-coded), and the CSD time-line trace (purple). Small colored dash to the left indicate a zero value for the FP generator. In general, CSD and FP-generator time courses match better than FP traces, but individual waves may still show strong differences in amplitude or even polarity due to addition or cancelation in CSD traces at instants of co-activation. N.a.u., p.a.u., normalized and proportional arbitrary units.

Adult female Wistar rats (>P60) were anesthetized with urethane (1.2 g/kg, i.p.) and placed in a stereotaxic device, and the body temperature was maintained at 37°C with a heating pad and feedback control. Glucose-saline was supplemented (10 ml/kg) every hour to maintain the animal hydrated, and experiments never lasted >4 h in total. The long-lasting anesthetic bupivacaine (0.75%) was applied at surgical wounds. In different experiments, concentric stimulating electrodes were placed in the soma layer of the CA3b region of the left hemisphere (AP 2.9; L ± 2.6; V 3.4 mm from Bregma and cortical surface) to activate the ipsilateral Schaffer input to CA1 and the commissural input to the contralateral CA1; in the angular bundle (AP 2.9; L ± 2.6; V 3.4) or the MEC (AP 2.9; L ± 2.6; V 3.4) to activate the medial perforant path (MPP); and in the lateral olfactory tract (LOT) (AP 2.9; L ± 2.6; V 3.4) to activate the lateral perforant path (LPP). Up to 64 simultaneous recordings were obtained with two linear silicon probes (32 sites, 65 μm intersite distance) from Atlas Neuroengineering (Leuven, Belgium). The probes were stereotaxically located at homotopic sites of the dorsal hippocampus across the CA1 region and that also spanned the DG/CA3 (AP 4-4.5; L ± 2.6 mm) (Figure 1A). Typically, the recording arrays also spanned the overlying V2 cortex and sections of the underlying thalamus. Mineral oil was used to cover the site of implantation after electrode penetration. Probes were soaked in DiI before insertion (Molecular Probes, Invitrogen, Carlsbad, CA) to assess their location post-mortem in histological sections. The built-in reference site was not used to avoid influence from nearby sources that severely distort the time course and spatial landmarks of local FPs. Instead, a silver chloride wire implanted under the skin of the neck served as a reference for recordings (note that volume-conducted contributions are readily separated by the ICA; see next section; Torres et al., 2019). Signals were amplified and acquired using MultiChannel System (Reutlingen, Germany), or Open Ephys hardware and software at a 50 kHz sampling rate. We used histological and electrophysiological criteria to identify cortical and hippocampal strata, such as the maximum of the population spike in evoked potentials, and the spontaneous cell firing to determine the position of cell layers in the CA1 and DG. At the end of the recording session the animals were sacrificed by anesthetic overdose, and their brain was removed and maintained in 4% paraformaldehyde in saline. Sagittal brain sections (100 μm) were stained with bis-benzimide and the electrode positions assessed by fluorescence microscopy.

### Signal treatment and analysis

Wide-band FPs (0.1 Hz–5 kHz) were recorded in 3 min periods separated by 15 min, and additional high-pass digital filter was set at 0.5 Hz to remove slow transient artifacts. Signals were down-sampled at 4 kHz to speed up analysis. Occasionally, the activity in a single recording site with transient artifacts was interpolated from the surrounding electrodes. We note that site interpolation in faulty electrodes does not affect the time-course of FP-generators extracted from a collection of recordings by the ICA (Herreras et al., 2015). Faulty recordings in outer sites were however rejected.

#### Spatial discrimination of intracerebral sources by ICA of FPs

Co-activation of converging pathways causes the respective FPs to mix linearly in the volume and contaminate each other. Therefore, we performed source separation through a blind source separation technique as the ICA (Bell and Sejnowski, 1995; Makarov et al., 2010; de Cheveigné et al., 2013; Glabska et al., 2014). The linear arrays are placed such that they span the volume occupied by the sources so that the obtained components resolve the electrical fields generated by different afferent pathways even when these make contact in the same neuron population as long as they do not contact identical postsynaptic territories. The pathway-specificity of most components returned by the ICA has been proved elsewhere through a battery of experimental tests and further supported by realistic feed-forward simulation of synthetic blends of FPs (Fernández-Ruiz et al., 2012; Martín-Vázquez et al., 2013; Torres et al., 2019). This enables several synaptic pathways to be explored simultaneously using a single recording track (Figure 1B). We thus refer to ICA components as FP sources or generators. Unlike the poor performance of ICA algorithms on surface EEG recordings, their optimal performance on intracranial multisite FPs derives from the fact that different synaptic pathways raise distinct voltage gradients in space, which can be captured by high-density recording arrays placed in or near the source populations (Figure 1C). Such pathway-specific voltage gradients fade and equalize in the distance, and cannot be discriminated by remote recordings (Herreras et al., 2023).

The ICA considers recorded FP signals *u_*m*_(t)* as the weighted sum of the activities of *N* neuronal sources or FP-generators:


(1) $$u_{m}\,\left(t\right)=\sum_{n=1}^{N}V_{m\,n}\,s_{n}\,\left(t\right),m=1,2,\ldots \,M$$

where (*V*_*mn*_) is the mixing matrix composed of the so-called voltage loadings or spatial weights of *N* FP-generators on *M* electrodes and *s_*n*_(t)* is the time course of the *n-th* FP-generator. Thus, the raw FP observed at the *m-th* electrode tip is a linear mixture of the electrical activity of several independent FP-generators. Using *u_*m*_(t)* the ICA finds both *(V_*mn*_)* and *s_*n*_(t)*. The joint group of spatial weights (*Vmn*) is ordered into instant depth profiles of the voltage according to electrode position. In the hippocampus, such curves match the spatial profiles of the standard evoked potentials of specific pathways (Figure 1D; Korovaichuk et al., 2010; Benito et al., 2014). Meanwhile the time-course *s_*n*_(t)* can be considered as a postsynaptic temporal convolution of spike output in an afferent population (i.e., afferent spike trains: Makarov et al., 2010; Figure 1E). The mathematical validation and practical limitations of this approach, as well as the possible sources of cross-contamination have been investigated thoroughly using realistic modeling of multisource FPs (for a review see Herreras et al., 2015). In this study we employed the kernel density ICA algorithm (Chen, 2006), customarily implemented in MATLAB (MathWorks). This algorithm outperforms other more common algorithms (e.g., infomax), particularly for signals with high rhythmic content (Fernández-Ruiz et al., 2013).

The analysis was performed with the LFP-sources^®^ software, freely available at http://www.mat.ucm.es/ṽmakarov/downloads.php. In former tests with synthetic FPs we found that the temporal fidelity of separated generators optimizes with the relative variance it contributes to the group of signals (Makarova et al., 2011). This enables different strategies to increase the relative variance of some FP generators, such as the selection of the recording channels making up the data matrix to optimize ICA separation of weak generators (Benito et al., 2014; Herreras et al., 2015). During irregular FP activity, different pathways vary their activation intensity with rapid dynamics, causing the relative instantaneous contribution of each to vary strongly with time. We previously found that this variability is optimal for ICA performance, although it favors maximum fidelity for the most powerful generators while the weakest ones get noisy (Makarova et al., 2011). Consequently, we set a minimum percentage of 1% relative variance in the analyzed period, otherwise they were rejected. Normally few LFP-generators (4-7) exhibited significant variance and distinct spatial distributions (Figure 1D), which permits further optimization by pre-processing the FPs prior to performing the ICA through dimension reduction using the principal component analysis (PCA). This approach efficiently diminishes the presence of noisy weak generators. The PCA also stabilizes and accelerates the subsequent convergence of the ICA.

For efficient separation of components, it is particularly important to reduce net time when two pathways co-activate tightly (Makarova et al., 2011). Such cases are revealed by unexpected humps in the spatial curves of returned components. Optimal separation can be pursued by repeating the analysis on epochs of increasing duration until the spatial profiles do not vary. In addition, spontaneous changes in the electrographic pattern from irregular to theta activity drastically altered the mean relative variance contributed by each generator, which may result in noisier extraction of FP generators. Since we are here interested in fine-grained temporal coherence we limited the study to irregular activity only with no theta epochs.

#### Current source density (CSD) of spontaneous FPs

The CSD is routinely used to remove the influence of sources located away from the recording area (volume-conducted potentials) (Lorente de Nó, 1947; Leung, 1979). We used the CSD here as a fast check for the visualization of the presence/absence of currents in a certain region or recording site. Assuming constant conductivity of the extracellular space σ we have: CSD = – σΔu, where u(t,x,y,z) is the electric potential and Δ is the Laplace operator. For linear probes with M recording sites (usually *M* = 32) we used a one-dimensional approach that calculates the CSD from the voltage distribution along the main cell axis:


(2) $$C\,S\,D_{m}\,\left(t\right)=-\frac{\sigma }{h^{2}}\,\left(u_{m-1}\,\left(t\right)-2\,u_{m}\,\left(t\right)+u_{m+1}\,\left(t\right)\right)$$

where *u_*m*_(t)* is the FP recorded at the *m-th* site and *h* is the inter-site distance. As it will be shown, the CSD time traces at a discrete point in space show notable differences when compared to the raw FP at that site, but they still contain mixed time courses of sources and sinks in the same site belonging to currents from different pathways with overlapped territories and own dynamics, hence they are unreliable and we rely on ICA for this purpose (Martín-Vázquez et al., 2013).

### Estimating the synchronization between FP-generators

Coarse synchronization between different recording sites and time courses of FP-generators was estimated using the cross-correlation coefficient (CC) and spectral coherence. The CC was obtained as:


(3) $$R=\frac{C_{12}}{\sqrt{C_{11}\,C_{22}}}\;$$

where (*C*_*ij*_) is the covariance matrix of two random variables. Spectral coherence was calculated by:


(4) $$C_{x\,y}\,\left(f\right)=\frac{{\left|P_{x\,y}\,\left(f\right)\right|}^{2}}{P_{x\,x}\,\left(f\right)\,P_{y\,y}\,\left(f\right)}$$

where *(P_*ij*_(f))* is the matrix of cross-power spectral density. To determine the level of significance we used the surrogate data test. Randomizing phase relations and keeping other first order characteristics intact, we obtained surrogate time series from the original signals. For each experiment we generated 1000 surrogates and we evaluated pairwise spectral coherences. The level of significance (at α = 0.05) was then calculated for each frequency value and coherence above this level was considered statistically significant.

We used two additional tests aiming at different features. Firstly, we use the *Pearson correlation coefficient*, a statistical measure used to quantify the linear correlation between two variables:


(5) $$\rho_{\text{XY}}=\frac{E\left[\left(X-\mu_{X}\right)\left(Y-\mu_{Y}\right)\right\}}{\sigma_{X}\,\sigma_{Y}}$$

which, applied to a sample {(*x*_*i*_,*y*_*i*_) : *i* = 1, …, *n*}, results in the following formula:


(6) $$r_{x\,y}=\frac{\sum_{i=1}^{n}\left(x_{i}-\bar{x}\right)\,\left(y_{i}-\bar{y}\right)}{\sqrt{\sum_{i=1}^{n}{\left(x_{i}-\bar{x}\right)}^{2}}\,\sqrt{\sum_{i=1}^{n}{\left(y_{i}-\bar{y}\right)}^{2}}}$$

The Pearson coefficient, when applied to two signals, can be used to quantify their degree of synchrony. This measure fluctuates in the interval [−1, 1], where absolute values close to 1 indicate a good correlation while values near 0 mean that the variables have no correlation at all. In our particular case we intend to study the variability in the coherence between right and left pairs of FP generators containing highly irregular fluctuations with patterns varying in a scale of seconds. It can thus be anticipated that the coefficient will vary along the time. Hence, we chopped the signals into equidistant time windows, and we repeated the analysis for three different values, Δt = 1, 0.1, and 0.01 s. The longer time windows will focus on the envelope of the signal in the low frequency range, whereas shorter ones will emphasize the correlation of faster fluctuations in the high frequency band. Choosing a single time window is troublesome as there are competing influences when signals as these are structured as evolving mixtures of long and short waves. Therefore, to help their interpretation we distributed the Pearson values into density histogram for each pair of signals compared and time windows, in order to better visualize if these correlation coefficients assemble around certain values of interest. The shorter the time window the more symmetrical and flat become the density histograms. Note these coefficients do not directly reflect frequency components or waves of a specific duration, albeit their presence in the signal has an influence.

Then, we used Granger-causality (Granger-c), a statistical test that is widely used to infer directional connectivity among brain areas (Chen et al., 2006). It assumes that, if there is a link from region X to region Y, then the past activity of X may predict the dynamics of Y. It is based on autoregressive models, where the time-course of the region Y is modeled by its own past and a residual (Bressler and Seth, 2011):


(7) $$Y\,\left(t\right)=\sum_{k=1}^{p}A_{k}\,Y\,\left(t-k\right)+\varepsilon_{Y}\;$$

Briefly, if there is a link from *X* to *Y*, the inclusion of the previous values of *X(t)* in the model of *Y(t)* would reduce the error of the prediction. Thus, the Granger-c from *X(t)* to *Y(t)* is determined by the F-statistics:


(8) $${ℱ}_{X\to Y}\equiv l\,n\,\frac{\left|\sum_{Y\,Y}\right|}{\left|\sum_{Y\,Y}^{\prime }\right|}$$

where Σ_*YY*_ = *cov*(ε_*Y*_) and $\Sigma_{Y\,Y}^{\prime }=c\,o\,v\,\left(\varepsilon_{Y}^{\prime }\right)$ are the residual covariance matrices of the model in (7) and the model including the past values of *X(t)*, respectively.

We employed the MVGC toolbox for Matlab to compute both temporal and spectral Granger-c (Barnett and Seth, 2014). As input time series, we used the activation signals of the right and left FP-generators (Dhamala et al., 2008). We downsampled the signals to 250 Hz and calculated Granger-c in a pairwise manner, using a sliding window of 5 s, with an overlap of 80% and a model order *p* = 12. The Granger-c associated to each link was the average value across time-windows.

To estimate the statistical significance associated to the Granger-c, we used a surrogate data analysis (*N* = 1000 in this work) by block-resampling. Each signal was cut at a single random time point and the blocks were permuted. By breaking the temporal relationship between signals, any connectivity between them would be by chance. For each surrogate, we kept the Granger-c values for the different links and approximated them to a normal distribution. We set the significance threshold at the value where the previous cumulative distribution was 0.95 (*p* = 0.05).

### Retrieval and quantification of Schaffer gamma waves

In order to compare the features of individual gamma waves and evaluate their synchronization and relative amplitude at left and right sites, accurate determination of the waveforms is required (start time, amplitude and duration). Here we used a formerly implemented method using deconvolution of FPs. The method employs the modeling of the time course of a FP generator, *s*(*t*), as a weighted sum of *K* single FP events of the pulse-like form:


$$s\,\left(t\right)=\sum_{k=1}^{K}w_{k}\,f\,\left(t-\tau_{k};\delta_{k}\right),f\,\left(t;\delta \right)=H\,\left(t\right)\,\frac{t}{\delta^{2}}\,e^{-t^{2}/2\,\delta^{2}}\;\left(9\right)$$

where *w*_*k*_, τ_*k*_, δ_*k*_ are the relative weight, starting time, and time scale of the *k-th* event, respectively, and *H(t)* is the Heaviside step function. We aim at estimating the parameter set (*w*, τ, δ) from the observation of *s(t)*. To accomplish this task, we use the method of maximization of the loglikelihood (for details see Benito et al., 2016). Events were considered paired when they overlap for at least 70 % of the duration. This procedure was also essayed on generators other than the Schaffer, but the results were unsatisfactory. This was partly due to fluctuating baselines and partly due to the imprecision of separated generators from FPs where multiple gamma oscillations are extensively overlapped in space (Makarova et al., 2011).

## Results

### Establishing homogeneous conditions for the analysis of irregular FP activity

Layer-specific FP motifs and oscillations spanning several contiguous electrodes in linear arrays are observed in all hippocampal subfields with multiple frequencies and durations (Figure 1C). These two spatial and temporal features reflected, respectively, the stratified input of various synaptic pathways to major populations of pyramidal or GCs, and the wide variety of dynamics. According to previous research (Herreras et al., 2022, 2023), such variety of waveforms derived in part from the spatiotemporal mixtures of potentials contributed by various co-active sources that contaminate each other and distort the original time courses. Temporal distortion arose either from the overlapping of the spatial domains hosting different FP waves/motifs (Figure 1C, purple ovals) or due to their contamination by others generated at remote sites (waves outlined in brown). Therefore, an ICA was employed to retrieve clean time courses of activities in separated sources in each hemisphere (Figures 1D, E). This approach exhibits a notable improvement over the conventional CSD analysis (Figure 1F), which removes remote contributions but does not separate waves generated by overlapping pathways (Martín-Vázquez et al., 2013). Thus, single-site CSD plots (Figure 1G, purple traces) still showed distorted waves when sources and sinks from different pathways cohabited the same strata. As described in Methods, only a few FP generators contributed significantly to the total variance of the epoch and were highly stable and reproducible across animals. The six main FP generators explored were the CA3→CA1 Schaffer input (Figure 1D, cyan), input to st. lacunosum-moleculare (st. L-M; green), MEC→DG input (red), the LEC→DG input (blue), input to GC soma layer (GCsom; magenta), and a remote FP generator (brown). The identification of these components was accomplished through the recognition of their characteristic voltage profiles (V_prof_) in conjunction with functional assessments, as reported in previous studies using selective stimulation of principal extrinsic and associational pathways (see below).

**FIGURE 2:**
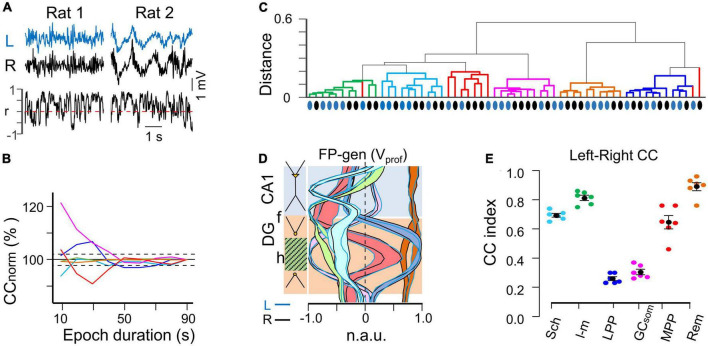
Spatial and temporal stability of homonymous bilateral FP generators. **(A)** Irregular FP activity may display different electrographic patterns (rat#1 or rat#2). The overall stability of fluctuations in the linear correlation was taken as a minimum criterion to ensure that changes of electrographic state did not affect measurement of the bilateral relationships. **(B)** The relative contribution of different generators to the raw FP may vary in a seconds-long time scale that is suboptimal for efficient segregation by the ICA. A minimum safe epoch duration was estimated by step-wise increase of the epoch until the cross-correlation (CC) between bilateral FP generators remained within 3% of the mean. **(C)** Hierarchical clustering of the spatial curves (Voltage profiles) for all generators across the animal population in both hemispheres (blue and black dots denote curves from the left and right hemispheres, respectively). With a few exceptions a distance < 0.2 grouped the profiles from different animals and hemispheres according to known anatomo-functional features. **(D)** Population spatial profiles for FP generators. The black and blue outlines correspond to the standard error of the mean power at each recording site. Note the close superposition of left and right profiles. **(E)** Population stability of bilateral CCs for FP generators during irregular activity. The colored dots represent individual experiments while the black dots reflect the mean ± s.e.m.

Left-right asymmetry was visually observed in raw FPs in certain spatial domains during epochs of irregular activity, which were the focus of our study. These epochs were characterized by fast activity of moderate amplitude (0.1–1 mV) which included interspersed slow waves in the st. L-M and occasional sharp waves (SPWs) within the st. radiatum of the CA1 area, commonly referred to as non-theta state. Given the known association between irregular FP states and diverse behaviors (Buzsáki et al., 1983), we conducted a preliminary evaluation to determine the relevance of sub-states with or without visually recognizable electrographic features (e.g., Figure 2A, rats #1 and #2), for bilateral comparisons. Specifically, we compared 10-second epochs (three per animal) at left and right homotypic sites in two demarcations: the st. L-M and the hilus. We calculated the mean linear correlation (Pearson coefficient) across the sample, yielding *r* = 0.55 ± 0.01 and *r* = 0.25 ± 0.01, respectively (mean ± s.e.m; *n* = 6 × 3; Δt = 0.1 s). Although there were different *r* values at different recording sites, there was very low variability across animals, regardless of the general pattern. Hence, no additional classification into raw FP electrographic sub-states was attempted.

However, due to the high short-term variability observed in the linear correlation (Figure 2A) it was deemed necessary to establish a minimum epoch duration during which the overall CC remained stable for all FP generators. To determine this, we gradually increased the epoch length until the CC between left and right homonymous FP generators did not deviate by more than 3% of the mean value observed in the shorter epochs (Figure 2B). Using this criterion, an epoch duration of 50 seconds was determined to be optimal under the present recording conditions. Thus, we analyzed three non-consecutive epochs per animal within a 60-minute period, and these epochs were bound together for subsequent analysis, resulting in a total epoch duration ranging from 50 to 90 seconds per animal.

### Spatial stability of bilateral FP generators

Field potential generators in left and right hemispheres were matched by their V_prof_. Hierarchical clustering analysis of all FP generator profiles obtained from the animal population (Figure 2C) revealed that homonymous left (blue dots) and right (black dots) FP generators clustered together within distances below 0.2, with some exceptions observed in the profiles of the MPP→DG generator (red), which contributed the smallest variance but still retained the main spatial landmarks. The tight spatial match of the left and right homonymous profiles was evident from the extensive overlap of the respective spatial bands outlining the standard error at each recording site (Figure 2D). This indicated a precise left-right symmetry of recordings, as displacements along the AP or LM axes rapidly modified the shape of the V_prof_ due to strong anatomical curvatures (Benito et al., 2014). Additionally, previous experiments have demonstrated that the spatial coherence of the MPP→DG generator drops the fastest of all FP generators, decaying rapidly for distances separated by more than 0.2 mm (Benito et al., 2014), which was used to estimate the interhemispheric symmetry of recordings and select the group of animals for study.

### Global correlation between homonymous bilateral FP generators is high in CA1 and low in the DG

As an initial global approach, we computed the overall CC for left-right pairs of homonymous generators (Figure 2E). The highest correlation was found for a remote FP generator originating in the overlying V2 cortex (as shown by the flat profile in brown in Figure 2D). The remaining generators were ranked in decreasing order of correlation as follows: L-M > Schaffer > MPP > LPP = GCsom. The first four generators had a CCmax greater than 0.6, whereas the last two in the DG have very low CCmax (<0.3). All correlations were found to be statistically significant at CCmax lag using a surrogate test (*n* = 1000 replications, α = 0.05) and none of the pairwise comparisons showed significant delays. The mean τ was (in ms, *n* = 6): 0.05 ± 0.15 (Sch), 0.1 ± 0.36 (L-M), −1.1 ± 1.6 (LPP), −0.6 ± 0.59 (MPP), 0.4 ± 0.48 (GCsom), and −0.7 ± 0.52 (Rem). Our analysis thus revealed no significant anticipation of global irregular dynamics in one hemisphere with respect to the other. It is important to note that these CC analyses were performed on wideband activities, meaning that no selection of frequency bands or individual waves was made at this stage. Further differences were observed through the fine-grained analyses detailed in the following sections.

### The Schaffer and L-M generators exhibit bilaterally coherent activity in the low-frequency bands, whereas only the Schaffer generator demonstrates coherence in the gamma band

**FIGURE 3:**
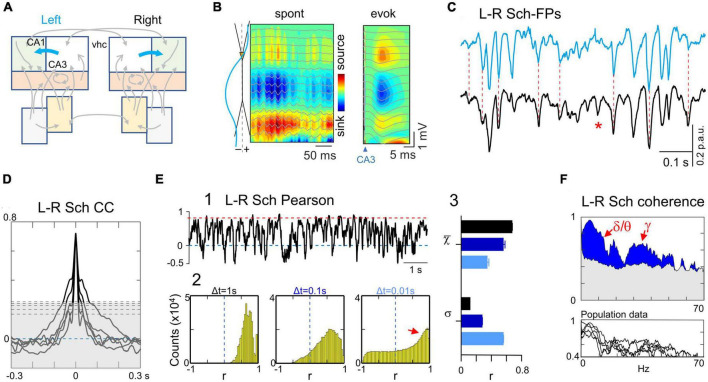
Tight bilateral synchrony of Schaffer-specific FPs. **(A)** Relevant pathways: CA3→CA1 Schaffer segments (cyan arrows). **(B)** CSD and superimposed FPs of a short spontaneous epoch containing a few gamma waves and a Schaffer-evoked subthreshold fEPSP. Spontaneous and Schaffer-evoked sources and sinks match the same spatial domains. Both types of potentials are selectively captured in the Sch generator returned by the ICA, whose spatial profile is shown to the left. **(C)** Instance of bilateral Schaffer-specific activities at homotypic sites. Note that Sch events exhibit a distinct negative-going fluctuation from a conspicuous baseline level. The left-right temporal match is nearly complete regardless of the amplitude and duration of waves, except a few that were unpaired (unilateral waves: red asterisk). **(D)** Crosscorrelograms of bilateral Schaffer activities were significant in all animals (surrogate test; *n* = 1000; horizontal dashed lines mark significance levels at 95% for each animal). **(E)** (1) The linear correlation (Pearson) shows sustained high value for long epochs (red dashed line) with abundant short-lived drops. (2) The fine temporal structure was explored by density histograms of r values at varying time windows. Even for Δt as small as 0.01 s the correlation index remained strongly positive (small red arrow). The shape and features of the distributions reflect temporal aspects of the correlation. It is expected that as Δt gets smaller the histograms become flatter and centered around zero (no correlation). The right-most portion (red arrow) at Δt = 0.01 s denotes abundant correlations of fast voltage fluctuations within this time-scale. Note however that these cannot be ascribed to waves in customary frequency bands as they may belong to waves of different duration with at least one fast limb. (3) The features of the density histograms were highly maintained across the animal population as noted by the narrow ranges of the mean (χ) and standard deviation (σ) (*n* = 5). **(F)** Spectral coherence showed significant bilateral matching (blue areas) across all main frequency bands, as illustrated in the upper and lower panels for a representative experiment and the population, respectively. The gray shading indicates the statistically significant threshold (95%), which consistently observed across all animals (*ca*. 0.4; surrogate test; *n* = 1000).

The Schaffer generator, which reflects spontaneous CA3→CA1 input (Figure 3A), exhibited a V_prof_ that closely resembled that of CA3-evoked potentials. It had a maximum at the CA1 st. radiatum and decayed toward both sides, the soma layer and distal apical dendrites. Additionally, a second, smaller peak appeared in the CA3 region, caused by coherent activation of CA3 axon collaterals that comprise the excitatory recurrent network (Martín-Vázquez et al., 2016). CSD contour plots during bouts of gamma waves closely matched subthreshold CA3→CA1 fEPSPs (Figure 3B). Notably, this generator is characterized by a conspicuous baseline on which gamma waves, SPWs, and other less stereotyped fluctuations grow in the same (negative) direction, denoting multiple firing regimes of the afferent CA3 population. The responsible synaptic pathway was functionally identified through selective capture of CA3-evoked potentials (Korovaichuk et al., 2010; Fernández-Ruiz et al., 2012). For estimating some quantifiers and statistics of activity, we manually removed the SPWs, as the large variance they contribute to the total epoch may bias comparisons.

In a representative experiment (Figure 3C) the tight left-right overlap of Schaffer activities was evidenced at different time scales and regardless of the amplitude of events. Therefore, all types of temporal motifs (gamma waves, SPWs or irregular waves) were highly coherent, despite the presence of some unpaired individual waves (asterisks). The left-right Schaffer CCs from all animals are presented in Figure 3D, all of which reached a significant level at CCmax lag (surrogate analysis, *n* = 1000; *p* < 0.05). This wide-ranging synchrony is best appreciated by the stable ceiling-like value of the Pearson’s linear correlation. However, we observed frequent short-lived drops of correlation, which we explored further by building density histograms using different time windows (Figure 3E). As Δt reduced from 1 s to 10 ms, we observed a gradual transition from a strong leptokurtic distribution with a large positive mean value to another markedly platykurtic but still with a positive mean value. The same trend was consistently repeated in all animals (Figure 3E, 3), which indicates tight bilateral correlation in both slow and fast fluctuations. These fluctuations belonged to temporally structured waves of stereotyped frequency, as denoted by the significant bilateral coherence found in spectral coherence analysis for delta/theta and gamma bands in all animals (Figure 3F, surrogate test, *n* = 400).

**FIGURE 4:**
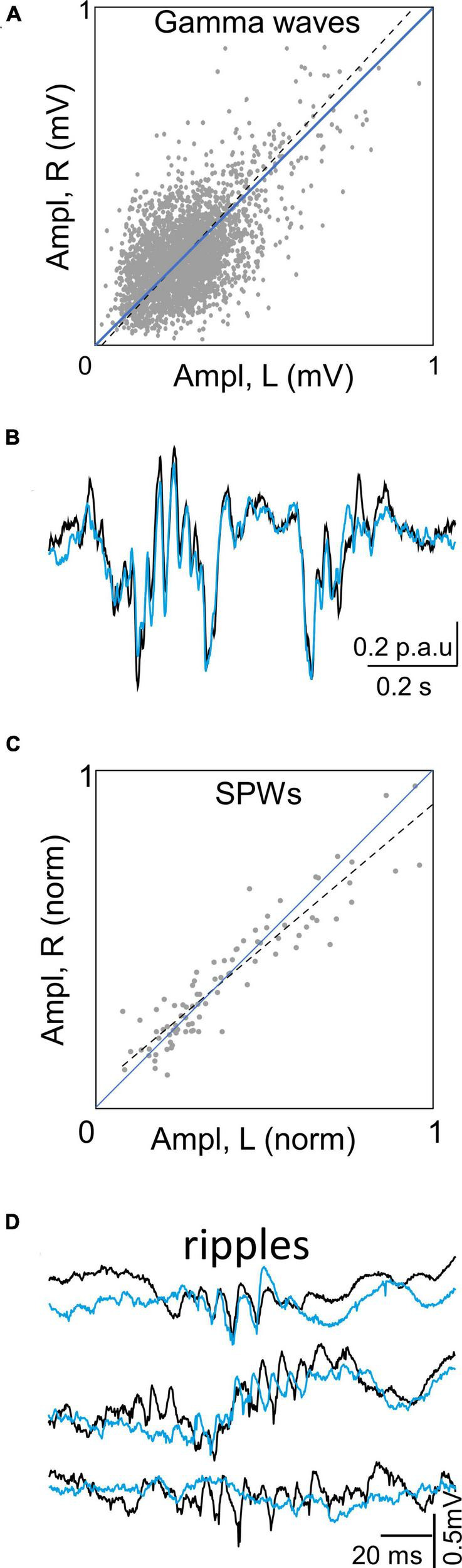
Bilateral covariation in Schaffer FPs depends on type of event. **(A)** Plot of amplitude of paired gamma waves in left and right sides. Gamma waves were extracted by a deconvolution method, measured in each side and considered as paired when the overlap was at least 70% of wave duration. The dos are values for individual pairs in a representative experiment. The large dispersion at both sides of the diagonal denote poor lef-right amplitude covariation. **(B)** Exemplary SPW superimposed in left and right sides. Note extraordinary overlap and covariation of composing wavelets. **(C)** Plot of amplitude covariation of SPWs found in all experiments (normalized to the largest). The little dispersion around the diagonal denotes tight left-right amplitude covariation. **(D)** Exemplary ripples recorded in the soma layer of CA1 during SPWs. Note overall coherence but discrepant and varying wavelet composition. Traces belong to raw FPs.

In former study we showed that the temporal characteristics of the Schaffer generator are optimal for extraction and quantification of individual gamma waves (Benito et al., 2016). Hence, we conducted a similar bilateral exploration in the present set of experiments (see Methods). The results confirmed tight matching of gamma waves but poor bilateral amplitude covariation of paired left and right gamma waves (Figure 4A). Bilateral SPWs showed, however, strong covariation (Figures 4B, C). Even composing wavelets were found to have extraordinary left-right covariation, distinguishing from baseline gamma waves. Thus, the larger and longer SPW events were bilateral in contrast to baseline gamma waves even though these were paired. Associated to SPWs, fast ripple oscillations were found to maintain global left-right coherence but the wavelet series were lateralized, could even fail on one side (Figure 4D). Note that these events do not belong to the Schaffer generator.

**FIGURE 5:**
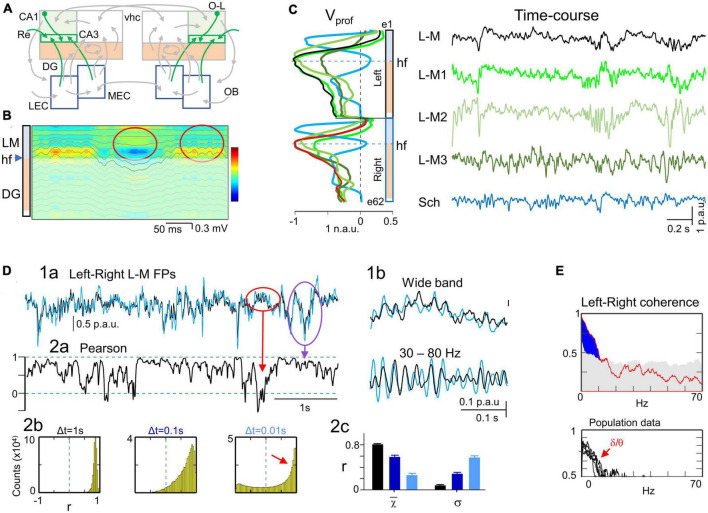
Bilateral synchrony of L-M FPs is restricted to low frequency delta and theta bands. **(A)** Relevant pathways: The st. L-M of the CA1 region receives numerous synaptic inputs in the distal apical dendrites of pyramidal cells, all of which are geometrically fit to raise FPs. Among others are the MEC, LEC, nucleus reuniens (Re) and several local inputs, such as or-lac interneurons (OL). **(B)** Reconstructed FPs and CSD from a sample epoch for the L-M generator. Note different types of slow and fast waves sharing the same domain with identical spatial profile whereas the corresponding source-sink pairs have inverted polarity (red ovals), which is indicative of different synaptic origins. **(C)** The characteristic profile of the L-M generator displays a single maximum within this stratum, which gradually decays toward the DG (voltage profile and time trace in black). The simultaneously obtained Schaffer component (cyan) is included for spatio-temporal comparison. To further disentangle the multiple pathways contained in the LM generator we constructed bilateral data matrices and applied a less stringent dimension reduction protocol. The ICA identified three bilateral components (LM1-LM3 in different shades of green) in all animals. These components exhibited spatial profiles that closely matched the one obtained for data in a single hemisphere (V_prof_ in black or red), allowing us to separate distinct L-M sub-generators. Although these sub-generators exhibit different dynamics, we retained the unified component for bilateral comparisons while we await characterization. **(D)** (1) The time course of the generic LM generator showed strong bilateral matching (1a), as indicated by a sustained high level of linear correlation over long periods (2a). However, brief periods of low correlation were also observed characterized by short-lived drops (compare red and purple ovals). (1b) The wideband and gamma filtered enlargements show that fragments of reduced bilateral correlation generally corresponded to poor phase matching of gamma waves. (2b) The fine temporal structure explored by density histograms of *r* values at varying time windows show similar trend as for bilateral Schaffer FPs (see small red arrow at Δt = 0.01 denoting abundant correlations of fast voltage fluctuations within this time-scale). (2c) The features of the density histograms were highly maintained across the animal population, as indicated by the narrow ranges of the mean and standard deviation (*n* = 5). **(E)** Bilateral spectral coherence showed significant values at low frequencies (delta and theta) whereas gamma reached no significance level in any animal.

The L-M FP generator (Figure 5A) presented V_prof_ with a maximum in the distal apical dendrites of the CA1, which reversed polarity in the st. radiatum, and decayed without reversal into the DG (Figure 2D). However, in CSD analysis (Figure 5B), the FP waves captured in this generator were found to be associated with both, source/sink and sink/source pairs in the same domain, which is consistent with former reports that found cohabitation of excitatory and inhibitory pathways in the st. L-M (Benito et al., 2014). Notably, the ICA was not able to discriminate waves with mirroring spatial profiles. As previously reported, the L-M generator captured theta rhythm when it is present. In such state, additional L-M generators were uncovered (Benito et al., 2014; López-Madrona et al., 2020), indicating that multiple inputs with similar profiles may remain unsegregated in the L-M generator. We essayed a formerly deployed strategy involving the modification of the spatial content of the data matrix (Benito et al., 2014) in order to enable the ICA algorithm for different spatial feature selection. We thus applied the ICA algorithm on a complete dataset of bilateral recordings and used less restrictive reduction of dimensions (see Methods). This procedure yielded three different components with similar (but not identical) spatial profiles in all animals (Figure 5C). All of these components had maxima at the st. L-M of the CA1 and exhibited different dynamics. Since they appeared twice in the spatial profiles of ICA components, one in each hippocampus (LM1-LM3), their respective bilateral activities must be highly coherent, otherwise each side would segregate into two different components, one per hemisphere, as it was the case in other generators (e.g., the LPP). However, we maintained the use of the compounded L-M generator for bilateral analysis due to the algorithm limitation for retrieving optimal dynamics in weak generators when using a single shank per hemisphere (Benito et al., 2014). Internal inconsistencies (over time) can now be explained by the composite nature, as for native FPs.

Upon examination of the generic L-M activity, it was observed that the baseline was less conspicuous compared to that of Schaffer FPs. This made it difficult to determine whether the positive and negative waves belonged to different pathways that were mixed in the L-M generator or if it was due to a zeroing effect caused by AC-filtered recordings. Previous studies (see Martín-Vázquez et al., 2013) have also noted this limitation. Nonetheless, at a coarse grain, the positive and negative waves were highly symmetrical (Figure 5D, 1a) in concordance with the high and sustained levels of linear correlation at any time windows (Figure 5D, 2). It is worth noting that drastic drops of linear correlation lasting from tens to a few hundred milliseconds (as evidenced by the red and purple ovals) corresponded to low-voltage epochs containing gamma oscillations. This was further confirmed at a fine grain scale by the poor and shifting phase matching of bilateral gamma waves in gamma-filtered traces (Figure 5D, 1b). Quantitative analysis showed the absence of significant bilateral coherence in the gamma band in spectral coherence analyses in all animals (Figure 5E; surrogate test, *n* = 400). In contrast, significant levels of coherence were observed for low frequency bands (<10 Hz).

### Ipsilateral Schaffer and L-M activities show no correlation at any frequency

**FIGURE 6:**
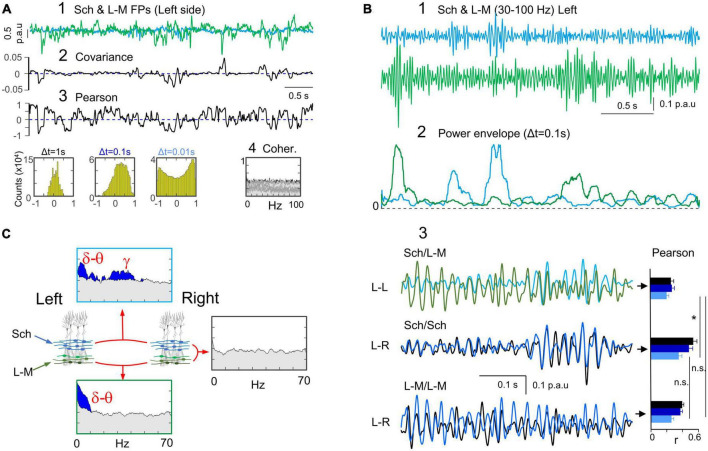
Ipsilateral Schaffer and L-M activities are unrelated despite some bilateral analogies. **(A)** (1) An overlay of a sample epoch of Sch and L-M activities in the left hemisphere indicates a near-complete lack of covariation between the two generators (2). (3) Winding excursions of the *r* values up and down from the zero indicate poor correlation, which was confirmed by the density histograms (near-zero mean value) for any Δt. (4) Spectral coherence analysis showed no significant frequency bands between these two generators in any animal. **(B)** Further analysis of Schaffer and L-M relationships within the gamma band (30–100 Hz). (1,2) Sample traces of the respective generators show independent occurrence of the larger bouts of gamma activity. (3) Superposition of gamma epochs in both generators (Sch/L-M) shown in greater detail and compared to bilateral overlays for each of them (Sch/Sch, and L-M/L-M). The simultaneous ipsilateral Sch and L-M traces reveal the phase inconsistency between gamma waves, as it was also the case for the left and right L-M generators. In contrast, the gamma waves from the Schaffer generators were almost completely matched in both sides. Note the different trend of the linear correlations with the variation of Δt (Pearson). Statistical comparisons are shown for a Δt = 0.01 s that is sensitive to rapid voltage fluctuations during gamma waves (Student’s *t*-test; *n* = 5; *p = 0.013). **(C)** Summary of ipsi and bilateral relationships of the Sch and L-M generators.

Given that the characteristics of wide-band bilateral correlation of the Schaffer and L-M activities were similar, and both reflect population synaptic inputs to the same CA1 pyramidal cell population, we sought to investigate possible relationships between their respective time courses in the same hemisphere. In sample epochs (Figure 6A, 1), the dynamics of these two generators displayed high levels of divergence. While the covariance was close to zero at all the times (Figure 6A, 2), the linear correlation showed strongly fluctuating positive and negative values (Figure 6A, 3), which is also indicative of a lack of correlation. This was further confirmed by the highly symmetrical *r* values around zero in the density histograms. However, a notable exception was observed for Δt = 0.1, which demonstrated a right-shift of the mean (positive correlation). This later result was stable across the animal population in both hemispheres (*r* = 0.06 ± 0.01, 0.24 ± 0.03, and 0.15 ± 0.03 for Δt = 1, 0.1, and 0.01 s, respectively; *n* = 10, data pooled from 5 animals and 2 sides). Moreover, we found no indication of lagged correlation between the Sch and L-M generators. The pairwise CC between Sch and L-M yielded a mean of 0.24 ± 0.03 and a mean T_max_ of −73.3 ± 40.4 ms, which did not reach significant level in any animal and hemisphere (surrogate test, *n* = 1000). Additionally, spectral coherence did not show significant values at any frequency band, animal, or hemisphere (Figure 6A, 4; surrogate test, *n* = 400 each).

We conducted further investigation into the possible relationship between Schaffer and L-M inputs, with a focus on gamma oscillations that may have been overlooked in wideband analysis. To achieve this, we bandpass-filtered FP generators in the 30–100 Hz range. The mean frequency of gamma-enriched time-courses (30–100 Hz bandpass) estimated on autocorrelation functions was 35.7 ± 1.2 Hz for the Schaffer generator and 37.3 ± 1.3 for the L-M generator. High-gamma (≈ 71 Hz) can also be disclosed in both by further narrowing of the bandpass filter (60–100 Hz). Visual inspection of sample epochs revealed that Sch and L-M gamma oscillations with amplitude above the baseline were not correlated, whether in long episodes or short bouts of gamma waves (Figure 6B, 1, 2). Close examination revealed that individual gamma waves were variably out-phased between the two generators (Figure 6B, 3; *r* = 0.2 ± 0.03, *n* = 5; Δt = 0.01 s), consistent with previous findings in bilateral comparisons of the L-M generator (*r* = 0.26 ± 0.03). Spectral coherence analyses between the two generators did not show any significant results for any frequency band. These results contrasted with the tightly matched bilateral gamma waves observed in the Schaffer pathway. Figure 6C provides a summary of the inter- and intra-hemispheric relationships between the two CA1 generators. Both generators showed strong bilateral coherence in the delta and theta bands but were uncorrelated between them, while only the Schaffer generator maintained strong bilateral gamma coupling. However, we cannot fully guarantee bilateral gamma decoupling in the L-M generator despite the current analysis, as we found it to be composed by a mixture of several pathways.

### Unveiling the asynchronous input from the lateral entorhinal cortices to DG generators

**FIGURE 7:**
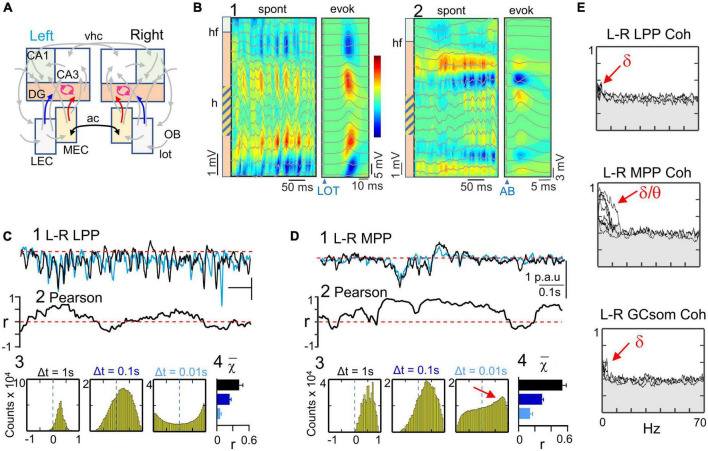
Bilateral asynchrony of the main cortical input from the lateral entorhinal cortices. **(A)** Relevant pathways: the three DG generators are highlighted in color. They all correspond to FP activities generated by the GC population: LEC (blue), MEC (red) and GCsom (fuchsia). The left and right counterparts of the MEC are connected through the anterior commissure (ac, black arrow), but there is no significant interhemispheric connection between LEC areas. **(B)** (1,2) CSD and superimposed FPs of two short spontaneous epochs across the DG containing bouts of alfa and gamma waves. To the right are the CSD and evoked potential profiles used to identify each pathway by stimulating the LOT (disynaptic DG activation through the LEC) (1), or a site in the angular bundle (AB) that predominantly activates the MPP→DG input. The stimuli were subthreshold in the DG. The sinks of current (blue contours) adjust to the outer and middle layers of the st. moleculare, respectively. Spontaneous and evoked sources and sinks match the same spatial domains. 1 and 2 are from different experiments. **(C,D)** correlation analyses of bilateral homonymous pathways reflected in the LPP **(C)** and the MPP **(D)** generators. The sample traces (1) belong to the same epoch. Note the discrepant left and right time courses for the LPP generators. Pearson coefficient was low and fluctuating around zero for the LPP generator (2). Individual alfa waves rarely coincide in left and right sides, although bouts of waves were more correlated, as reflected in the right-ward shift of the density histogram at Δt = 0.1 s (3,4). On the contrary, the few waves appearing in the MPP generators were tightly matched, which was reflected in the density histogram (red arrow at Δt = 0.01 s) (3). Pairwise comparison of the histogram population values (4) between LPP and MPP were all significantly larger for the latter (*t*-Student, *n* = 5 each; *p* ≤ 0.0001 in all cases). **(E)** Left-right spectral coherence for all experiments and DG generators. The LPP and the GCsom generators showed a small but significant coupling at delta frequencies, while the MPP activities displayed significant coherence in frequencies up to approximately 10 Hz (surrogate analysis; *n* = 400).

The ICA of FPs in the DG subfield yielded three distinct components with variance greater than 5%, indicating their stability (Figure 7A). These components displayed voltage profiles with higher values and positive-going fluctuations in the hilus between DG cell layers, but differed in the location of polarity reversal (Figure 1D). Raw FP waves with other spatial configurations were observed, but were not included in the study due to insufficient cumulative variance. The stronger and the weaker components were identified as the LPP and MPP generators, respectively, as they captured subthreshold evoked potentials elicited by stimuli in the LOT di-synaptic activation input to the DG through the LPP and the MPP pathways, respectively. Figure 7B displays FPs and CSD of a sample epoch, as well as the evoked potentials for each generator, revealing source/sink pairs that correspond tightly in space with those of spontaneous waves, with sinks in the outer or the middle molecular layers of the DG, respectively. They thus reflect the ongoing separate input from the LEC and MEC to the GC population. The third generator, GCsom, corresponds to inputs at the granule-cell layer and proximal dendrites, and has been identified as a local generator whose dynamics closely follow that of the LPP generator (Benito et al., 2014).

In the present experiments, the LPP and GCsom generators exhibited alpha activity dominating over bouts of shorter (gamma) waves and irregular activity (Figure 7C). The MPP generator, on the other hand, displayed sparse low-amplitude activity and few large slow waves (Figure 7D). In both generators, a consistent baseline was observed, from which fluctuations departed in a unidirectional manner. However, gamma waves typically rode on slower potentials, which difficulted their separate extraction for precise wave-to-wave characterization.

The superposition of left and right activities in the LPP generator revealed strong mismatch (Figure 7C, 1), as evidenced by fluctuations up and down the zero value in left-right linear correlation, (Figure 7C, 2), even during long epochs of simultaneous presence of gamma waves, which indicated hazardous bilateral phasing of individual waves. The corresponding density histograms displayed small right-ward shifts at Δt ≥ 0.1 s, but were symmetrical for Δt = 0.01 s (Figure 7C, 3), indicating moderate synchronization of slow fluctuations but little in faster ones. These values were highly reproducible across the population (Figure 7C, 4). In contrast, the MPP generator showed tight left-right matching of their activities (Figure 7D, 1), resulting in higher values of correlation (Figure 7D, 2), and clear right-ward drift in density histograms for any Δt (Figure 7D, 3). These values were also highly reproducible across the population (Figure 7D, 4), and significantly larger than those for the bilateral LPP (*p* = 0.02 or smaller, *n* = 5; Student-*t* test). Spectral coherence for the bilateral LPP generators showed minimum significant bars limited to the delta band in 3 out of 5 experiments (Figure 7E) (surrogate analysis, *n* = 400). Similar findings were obtained for the GCsom generator. Conversely, the MPP generator showed significant coherence in all animals spanning the delta and theta bands, but not in the gamma band.

### Granger-test reveals some ipsilateral but not bilateral leading FP generators

**FIGURE 8:**
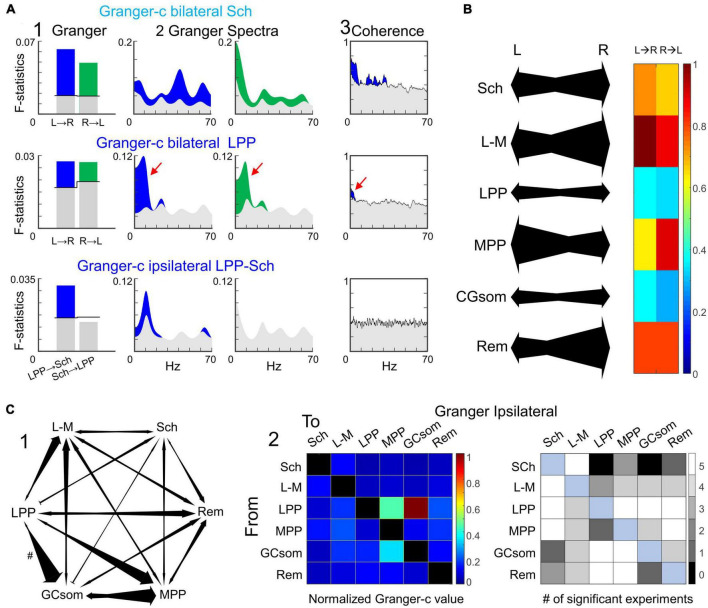
Granger-c testing shows no directionality between homonymous FP generators but it is present in some ipsilateral pairs. **(A)** Representative examples of one experiment for three relevant pairs. Panels 1 and 2 show the temporal and spectral Granger-c, respectively, and panel 3 the spectral coherence associated to each pair. Color-coded sections are the values above the significance threshold (gray values). No clear hemispheric leadership was found. In bilateral tests (upper and middle rows), there was a bidirectional connectivity between hemispheres at generator-specific frequencies that agreed with the spectral coherence (red arrows). The ipsilateral analysis revealed some clear directionalities. The example of the third row shows the unidirectional link from LPP to Schaffer, with no connectivity in the opposite direction. **(B)** On the left, connectivity values of a representative experiment. The size of the arrows represents the intensity of the connectivity. On the right, average values across the population of temporal Granger-c for each homonymous pair. While the links were not completely symmetrical, there were no significant differences between directions across subjects. **(C)** Ipsilateral analysis of Granger-c. (1) Example of the ipsilateral connections for one experiment. The link from LPP to GCsom (#) has been halved scaled. (2) The left panel represents the average Granger-c for each pair, and the right panel depicts the number of subjects with a significant connectivity value. The FP generator from lateral entorhinal cortex (LPP) has a leading role in the connectivity, mainly propagating to MPP and GCsom.

To check for possible leadership of the synaptic pathways represented in FP generators in one side respect to the other we essayed the Granger-causality (Granger-c) between pairs of right and left homonymous generators. Figure 8A (1) shows two instances of temporal Granger-c in a representative experiment (upper and middle rows) and along the frequency spectrum in the two directions (2). The significant frequencies (over the gray profiles) in such spectra were largely but not completely coincident between directions (e.g., upper row). Nearly all pairwise comparisons (57 out of 60 comparisons: 5 animals x 6 generators x 2 directions: Supplementary Table 1) yielded statistically significant value (surrogate test, *n* = 1000), and we found no clear leading of one side respect to the other. The net value was, however, generator-specific (mean population value is color coded in Figure 8B along a diagram representation of directionality), and it followed the general trend found in precedent tests, that is, high between all homonymous pairs except the LPP and GCsom generators that showed weaker value (compare significant bars in Granger-c and spectral coherence plots: Figure 8A, 3).

We also essayed the Granger-c test on pairs of generators in the same hemisphere. The results were more varied than interhemispheric correlations (Figure 8B, 1), and numerous directional relations were found as well as non-significant pairs in either direction. Except in the case of the LPP generator that showed the strongest directionality over all other generators, the Granger-c indices were weak and reached statistical level in only a fraction of the animals tested (plot in gray squares). The results in Figure 8C correspond to ipsilateral comparisons in the left side, and were largely replicated in the right side.

## Discussion

The spatial discrimination of components in bilateral FPs within the hippocampus enables access to the ongoing temporal dynamics of certain afferent populations and internal pathways in both hemispheres. We found that, in the non-theta state, these pathway-specific activities, or FP generators, displayed highly distinct dynamics, yet maintained a global bilateral coherence that was more pronounced for slow waves, but barely noticeable for shorter gamma waves. The main input to the hippocampus from the LEC was a notable exception, as it was strongly asymmetric. We propose that the lateralized activity entering from the LEC is synchronized in homonymous pathways of both hemispheres for integration purposes across interhemispheric projections, while the bilateral decoupling of gamma waves indicates their preferential involvement in individual ipsilateral pathways.

The nature of field potentials (FPs) is predominantly synaptic (Elul, 1971; Haberly and Shepherd, 1973). Most FP generators have been identified previously (Sch, LPP, and MPP), while others have been associated with activity in populations of local interneurons (GCsom), or remained as a group of pathways that ICA cannot easily separate (L-M) (Benito et al., 2014). Here, we show that this latter generator comprises at least three different synaptic pathways with own dynamics that make synaptic contact in the same distal apical dendritic domain of CA1, but their identification is pending. The anatomical candidates that meet this requirement are the nucleus reuniens, LPP and MPP, O-LM interneurons, and neurogliaform cells (Wouterlood et al., 1990; van Groen et al., 2003; Somogyi and Klausberger, 2005; Vertes, 2015; Goswamee et al., 2021). Taken together, the FP generators studied here are far from providing a complete map of the connectivity of the cortico-hippocampal circuits. Actually, they belong to the few connections that contribute significantly to FPs. In former studies we showed that most pathways do not contribute to FPs, either due to the inadequate spatial geometry of microscopic currents (Herreras et al., 2023) or because the low level of activity does not accumulate enough variance to be discriminated by the ICA (Herreras et al., 2015). However, in these loose pieces of the map, we have several that are key and allow us to glimpse the overall flow of activity and some of its bilateral characteristics.

### Technical issues

Comparisons of population activity in neuron populations are traditionally performed by FPs in the frequency domain. The uncontaminated temporal fluctuations of FP generators allow for more secure comparisons in the time domain. Prior studies using realistic feed-forward models of FPs have demonstrated that while FPs are formed from elementary synaptic currents of relatively stable duration, their scaling up to mesoscopic FPs give raise to waves of variable length when combined in different temporal patterns, which enters energy in different frequency bands (Makarova et al., 2011). As such, their interpretation should be approached with caution as they do not directly correspond to true elementary waves or their intervals. To minimize over-interpretation by relying on a single aspect (Hillebrand et al., 2012; López-Madrona et al., 2017), we have utilized several tests exploring different temporal, frequency, and leadership characteristics.

The ICA has been successfully used to explore different aspects of FP mixtures, either in the spatial or the temporal and frequency domains (Makarov et al., 2010; Schomburg et al., 2014; Lopes-Dos-Santos et al., 2018; Rogers et al., 2019). Demixing of irregular FP activity is well suited to ICA, but that of gamma oscillations is difficult in certain regions, such as the DG, especially during periods of high co-activation due to excessive spatial and temporal overlap. In previous computational studies, we showed that co-activation of more than three spatially distinct generators with a similar frequency results in a significant reduction in temporal accuracy by the ICA (Makarova et al., 2011). Methods to increase the temporal precision of separation have been advanced, but they may not achieve sufficient precision in some epochs (Benito et al., 2014, 2016). Without optimization measures, accurate extraction of gamma waves in strongly overlapping DG generators (e.g., Fernández-Ruiz et al., 2021) is highly susceptible to error, while being more reliable when applied to CA1 gamma sources (see Herreras et al., 2023 for a repertoire of gamma-related phenomena). In this study, we have opted for cautious use of correlation or spectral coherence tests, which are suitable for studying global flow and coherence between paired generators of bilateral circuits. It is possible to extract individual gamma waves for bilateral wave-to-wave comparison in exceptionally favorable generators such as the Schaffer generator (Figure 4; Benito et al., 2016). We have attempted to perform the same process on gamma waves from other generators in the DG, but the results have not been satisfactory, mainly due to the lack of an adequate baseline that allows precise estimation of time and amplitude parameters of individual waves.

### Different dynamics in ipsilateral generators denote nodal processing

The FP generators analyzed show very different dynamics, even though some of them are part of the canonical excitatory trisynaptic circuit (Andersen et al., 1971; Herreras et al., 1987), where some impact from one node or population to another might have been expected. The failure to do so implies that the dynamics of each segment results from the convergence of several synaptic afferents in each population, and would explain the small number of ipsilateral generator pairs showing directionality with the Granger-c test, confirming previous findings in awake rats (López-Madrona and Canals, 2021). Only the GCsom generator showed dynamics inherited from the LPP, as can be expected from an interneuron subsystem that receives few inputs, preferentially from its upstream excitatory population.

Theoretical studies have found that effective connectivity in the hippocampus can be strongly modulated by multiple factors, including the interaction between common afferents, average firing frequency, or intrinsic excitability (Battaglia et al., 2012; Han et al., 2015; López-Madrona et al., 2017; Pariz et al., 2018). On the one hand, experimental studies conducted on single subfields will be highly dependent on the study conditions, and on the other hand, theoretical studies show that unless structural and functional constraints are incorporated, almost any parameter can be decisive in one range or another (Migliore et al., 1995; López-Aguado et al., 2000; Ibarz et al., 2006). This is the reason why simultaneous experimental observation of several nodes is a necessary approach to first delineate the physiological ranges of activity in specific synaptic pathways.

Changes in effective connectivity have already been observed within the hippocampus. A most remarkable case is the opposite modulation of excitability and neuronal firing rate in CA1 and DG upon changes from irregular to theta related behaviors, by which GCs augment firing rate whereas there is a drastic reduction of CA1 pyramidal cell firing (Leung, 1980; Buzsáki et al., 1983; Herreras et al., 1988; Vinogradova, 2001). The flow reversal occurs in the CA3 region that is capable to perform strong nonlinear transformations (Guzowski et al., 2004). This region is an important node where cortical and subcortical information converge, and is also the main hippocampal node of interhemispheric connection. The opposite behavior of two consecutive nodes in a circuit not only demonstrates it does not operate as synfire chains (feed-forward assembly-based transmission: Abeles, 1991), but also evidences that the output of a population may have little temporal correspondence with its inputs, mostly when it is engaged in performing specific logical operations, such as feature extraction, coincidence detection, or others. Despite the fact that entorhinal afferents contact all three hippocampal nodes (DG, CA3, and CA1) and bypass the canonical trisynaptic chain (Hjort-Simonsen and Jeune, 1972; Steward and Scoville, 1976; Ruth et al., 1982, 1988), each of these shortcuts can transmit different dynamics (Traub and Whittington, 2022). In this line, the different FP generator dynamics before and after CA3 reflects logical processing within specific nodes of the circuit.

### The frequency selective bilateral coherence of field potential (FP) generators may reflect preferred patterns for interhemispheric information exchange

Experimental studies with repetitive stimulation have shown that transmission through hippocampal circuits to the cortex is frequency-dependent (Andersen and Lomo, 1967; Herreras et al., 1987; Moreno et al., 2016). Optimal frequencies depend on the node used for stimulation, which indicate that different frequency filters could be sequentially imposed on successive nodes. Intuitively, this should end flow of activity after a few nodes. However, exogenous stimulation imposes frequency-dependent cumulative effects on single cells that do not take place during the sparse and scattered transmission of spontaneous activity. According to present data, spontaneous transmission appears to operate quite differently, as neither long nor short waves show coherence across ipsilateral FP generators.

Bilateral coordination may arise from external nuclei with bilateral projection to both hippocampi (e.g., raphe, medial septum, nucleus reuniens), or it may be established internally through direct interhemispheric (CA3-CA3, CA1-CA1), crossed (CA3-CA1), or indirect connections (DG-DG through hilar cells: Amaral, 1978). Interestingly, while the MEC and various retrohippocampal areas have reciprocal and crossed connections, the LEC lacks direct reciprocal connections (Steward and Scoville, 1976; Köhler et al., 1978; Amaral et al., 1984; van Groen and Wyss, 1990; Watson et al., 2017), which explains the independent activity of the left and right LEC generators. Given its position at the beginning of the cortico-hippocampal chain, this seems like a logical result given the considerable functional lateralization of the cortex in humans, as well as in rodents (Cohen and Wilson, 2017; Dieterich and Brandt, 2018; Bartolomeo and Seidel Malkinson, 2019).

A candidate for interhemispheric synchronization might come by the highly symmetric input from the MEC, which has strong reciprocal bilateral connections and may impact all nodes of the trisynaptic circuit (Blackstad, 1956; Köhler et al., 1978). Yet, its low level of activity under present conditions does not make it appropriate for acting as a coordinator of bilateral subfields. Instead, we consider node-by-node synchronization via direct or indirect (disynaptic) connections to be more likely. In this respect, it is notable that individual generators show different regimes of activity, displaying slow waves of varying duration interspersed with shorter highly stereotyped gamma waves. In a former study we showed that in the Schaffer segment a gamma wave corresponds to the synaptic envelope caused by synchronous firing of a CA3 assembly (Fernández-Ruiz et al., 2012), as also confirmed by realistic feed-forward modeling (Martín-Vázquez et al., 2013). Although the firing synchrony of the afferent neurons that elicit a gamma wave is stronger than that of the slower waves, these are much larger and therefore require the activation of a much larger pool of afferent neurons. Therefore, the synaptic envelope built on individual postsynaptic cells is more likely to reach threshold (Combe et al., 2018) and thus have a stronger impact on the contralateral side. For instance, SPWs are associated to strong recruitment of pyramidal cell firing in CA1 (Csicsvari et al., 1999) whereas this is smaller and very specific during gamma waves (Senior et al., 2008; Fernández-Ruiz et al., 2012). In fact, the bilateral synchrony of gamma waves in this segment is produced by coordination of autonomous interneuronal oscillators on each side (Bartos et al., 2002; Börgers et al., 2012; Benito et al., 2016), since it persists, although without bilateral pairing, after blockade of the commissure. In contrast, the longer and larger SPWs disappear on both sides although they resume later, indicating their dependence on interhemispheric reciprocal excitation (Martín Vázquez and Herreras, 2015).

### Do left and right corticohippocampal circuits transmit different information despite of the bilateral coherence?

The different dynamics of FP generators in the same hemisphere indicates that FP timing does not reflect simple forward transmission. Rather it is compatible with specific processing at each node, and the lack of bilateral coherence for fast waves indicates different streams of information in each side. However, we only count with a few FP generators. We lack the GC output to CA3 population and the CA1 output to the subiculum. The first of these two has been found to be lateralized during processing of contextual navigational cues using calcium imaging of GC populations (Cholvin and Bartos, 2022). This result is compatible with the LEC inputs differing in left and right sides we found here. Experiments to check if lateralization of the LPP maintains in behaving animals are on the way in our lab.

In turn, the wave-to-wave bilateral gamma coupling of the CA1-CA3 Schaffer generator appears to be an exception to the lateralized dynamics of gamma waves in the other FP generators. It seems to support that CA1 output at the end of the hippocampal chain has become bilateral on its way to the cortex. However, as previously shown and it is further confirmed in this study, the left and right Schaffer gamma waves have very low amplitude covariation, indicating that the neural assemblies responsible for each gamma wave in each hemisphere (Fernández-Ruiz et al., 2012) transmit essentially different information (Benito et al., 2016). Based on this, we infer that long (large) waves in the delta/theta band are likely to transfer information both ipsi and contralaterally while gamma waves would remain largely ipsilateral. Congruent with this view is that SPWs match bilaterally much more precisely than gamma waves in the same generator. Even, other faster waves that could be expected to match bilaterally as the ripples, are moderately coherent but poorly matching wave-to-wave (Villalobos et al., 2017; this work).

## Conclusion

The main inputs to the hippocampus from the LEC and MEC reflect different types of information and are involved in specific behavioral subtasks that require coordination (Boisselier et al., 2014; Schlesiger et al., 2015; Wang et al., 2018). We envision the LEC as a bilateral anatomical node that collects and transmits lateralized information to the hippocampus, where it is parallelized by interhemispheric connections in specific nodes that require timing to effectively exchange data toward the completion of any of the multiple functions attributed to this system.

Furthermore, the different temporal dynamics between nodes within the same hemisphere, together with the bilateral symmetry in long-duration waves and the general decoupling of both intra- and interhemispheric gamma waves, are compatible with the idea that slow waves reflect moments of information flow that need to be parallelized in specific nodes, while fast waves reflect local ipsilateral processing. Anatomically, some entorhinal, perirhinal, and retrohippocampal areas have been shown to project ipsilaterally to some nuclei and bilaterally to others (Krettek and Price, 1977; Köhler et al., 1978; Wyss, 1981; van Groen and Wyss, 1990; van Groen et al., 2003). Hence the possibility of two systems, one ipsilateral and one bilateral cannot be discarded.

Taken together present and former results, it can be inferred that the synchrony or lack thereof displayed in the bilateral FPs does not necessarily reflect redundant information flow in both hemispheres, but merely their timing. That is, symmetrical voltage fluctuations do not imply identical replicas in left and right sides. On the contrary, they suggest a system of node-by-node comparison and/or selective information extraction that continues to flow separately in both hemispheres. In this essentially lateralized processing that we are proposing, the LEC would play an important role, projecting to numerous nuclei and cortical areas (Krettek and Price, 1977; Haberly and Price, 1978; Wyss, 1981).

We speculate that if bilateral projection was generalized over the brain, along with dense interhemispheric connections in the corpus callosum and the commissures, the susceptibility to hypersynchronous pathological entrainment would be more accentuated than it already is, and would also reduce the processing capacity, which is not particularly advantageous in organisms with axial symmetry that benefit from lateralized gathering of sensory information and independent control of the limbs.

## Data availability statement

The original contributions presented in this study are included in the article/Supplementary material, further inquiries can be directed to the corresponding authors.

## Ethics statement

The animal study was reviewed and approved by the Research Committee of the Cajal Institute.

## Author contributions

SH-R performed the experiments. SH-R, RM-A, and VL-M programmed and together with JM analyzed the data. SH-R and OH prepared the illustrations. OH designed the experiments and wrote the manuscript. All authors revised and approved the final manuscript.
