# Supplementary material for: Uncorrelated bilateral cortical input becomes timed across hippocampal subfields for long waves whereas gamma waves are largely ipsilateral
Source: Front Cell Neurosci. 2023 Jul 27;17:1217081. doi: 10.3389/fncel.2023.1217081 (PMC10412937; doi:10.3389/fncel.2023.1217081)
Supplement: Supplementary file 1 [file Table_1.DOCX]

Supplementary Material

Uncorrelated bilateral cortical input becomes timed across hippocampal segments for long waves whereas gamma waves are largely ipsilateral

Sara Hernández-Recio, Ricardo Muñoz-Arnaiz, Víctor López-Madrona, Julia Makarova, Oscar Herreras

*** Correspondence:** Oscar Herreras: herreras@cajal.csic.es

# Supplementary Table 1. Bidirectional values of Granger causality (Gc) test for left (L) and right (R) FP generators. Each column corresponds to a different animal. Values in black are above significant level (Surrogate test; n=1000), and those in red were non-significant. Sch, Schaffer; L-M, lacunosum-molecualre; LPP, lateral performant pathway; GCsom, Granule cell soma; MPP, medial performant pathway; Rem, remote generator (volume-conducted from cortex). The normalized mean values are in Figure 8B.

|  |  | Rat 1 | Rat 2 | Rat 3 | Rat 4 | Rat 5 |
| --- | --- | --- | --- | --- | --- | --- |
| Sch | *Gc L → R* | 0.057 | 0.063 | 0.060 | 0.040 | 0.054 |
|  | *Gc R → L* | 0.049 | 0.049 | 0.035 | 0.061 | 0.061 |
| L-M | *Gc L → R* | 0.063 | 0.091 | 0.107 | 0.040 | 0.079 |
|  | *Gc R → L* | 0.072 | 0.058 | 0.046 | 0.118 | 0.041 |
| LPP | *Gc L → R* | 0.034 | 0.028 | 0.029 | 0.019 | 0.030 |
|  | *Gc R → L* | 0.027 | 0.027 | 0.024 | 0.026 | 0.026 |
| GCsom | *Gc L → R* | 0.033 | 0.031 | 0.032 | 0.022 | 0.021 |
|  | *Gc R → L* | 0.027 | 0.027 | 0.017 | 0.018 | 0.021 |
| MPP | *Gc L → R* | 0.021 | 0.052 | 0.093 | 0.042 | 0.034 |
|  | *Gc R → L* | 0.044 | 0.078 | 0.072 | 0.089 | 0.061 |
| Rem | *Gc L → R* | 0.087 | 0.083 | 0.049 | 0.038 | 0.048 |
|  | *Gc R → L* | 0.057 | 0.042 | 0.075 | 0.083 | 0.046 |
